# Higher fine-scale genetic structure in peripheral than in core populations of a long-lived and mixed-mating conifer - eastern white cedar (*Thuja occidentalis *L.)

**DOI:** 10.1186/1471-2148-12-48

**Published:** 2012-04-05

**Authors:** Madhav Pandey, Om P Rajora

**Affiliations:** 1 Canadian Genomics and Conservation Genetics Institute, Faculty of Forestry and Environmental Management, University of New Brunswick, 28 Dineen Drive, Fredericton, NB E3B 5A3, Canada; 2 Current address: Department of Plant and Soil Science, Texas Tech University, 15th and Detroit Avenue, Lubbock, Texas 79409, USA

## Abstract

**Background:**

Fine-scale or spatial genetic structure (SGS) is one of the key genetic characteristics of plant populations. Several evolutionary and ecological processes and population characteristics influence the level of SGS within plant populations. Higher fine-scale genetic structure may be expected in peripheral than core populations of long-lived forest trees, owing to the differences in the magnitude of operating evolutionary and ecological forces such as gene flow, genetic drift, effective population size and founder effects. We addressed this question using eastern white cedar (*Thuja occidentalis*) as a model species for declining to endangered long-lived tree species with mixed-mating system.

**Results:**

We determined the SGS in two core and two peripheral populations of eastern white cedar from its Maritime Canadian eastern range using six nuclear microsatellite DNA markers. Significant SGS ranging from 15 m to 75 m distance classes was observed in the four studied populations. An analysis of combined four populations revealed significant positive SGS up to the 45 m distance class. The mean positive significant SGS observed in the peripheral populations was up to six times (up to 90 m) of that observed in the core populations (15 m). Spatial autocorrelation coefficients and correlograms of single and sub-sets of populations were statistically significant. The extent of within-population SGS was significantly negatively correlated with all genetic diversity parameters. Significant heterogeneity of within-population SGS was observed for 0-15 m and 61-90 m between core and peripheral populations. Average *Sp*, and gene flow distances were higher in peripheral (*Sp *= 0.023, σ_g _= 135 m) than in core (*Sp *= 0.014, σ_g _= 109 m) populations. However, the mean neighborhood size was higher in the core (*Nb *= 82) than in the peripheral (*Nb *= 48) populations.

**Conclusion:**

Eastern white cedar populations have significant fine-scale genetic structure at short distances. Peripheral populations have several-folds higher within-population fine-scale genetic structure than core populations. Anthropogenic disturbances and population fragmentation presumably have significant effects on fine-scale genetic structure in eastern white cedar. Core populations have higher neighborhood size than peripheral populations, whereas gene flow distances are higher in peripheral than in core populations. The results of our study contribute to the knowledge of poorly-understood spatial genetic structure of core versus peripheral populations in plants. As well, the information is of significance for conservation of genetic resources of eastern white cedar and perhaps of other long-lived forest trees with mixed-mating system.

## Background

Recently, in addition to core, peripheral populations are also gaining importance for gene conservation, because under global climate change conditions they may possess genotypes of future adaptive potential. Plant populations tend to migrate further from their range periphery to ensure their survival by developing adaptability in a changing environmental condition due to global warming [1]. Genetic studies involving both core and peripheral populations are still limited [2]. Additionally, the available studies mainly focused on within-and among-population genetic diversity [2-4], and majority of them reported higher genetic diversity and lower population differentiation in core than in peripheral populations. Although still limited, there are increasing reports on SGS in fragmented versus continuous populations e.g., [5-7]. However, information on the comparison of SGS between core and peripheral populations of plant species is almost non-existent. The only published report on this aspect by Gapare & Aitken [8] reported significantly higher SGS in peripheral and random distribution of genotypes in core populations of predominantly outcrossing conifer, Sitka spruce (*Picea sitchensis*). The level of SGS within plant populations depends on seed and pollen dispersal, mating system, mode of reproduction, size of the founder population, species longevity, adult population density, effective population size, and micro environmental selection [9-13]. Anthropogenic disturbance and fragmentation of populations can also influence SGS by restricting gene flow and increasing local drift due to severe modification of forest environment and ecosystem, and density of populations [14,15]. Information on SGS levels in core and peripheral populations can unravel differential effect of the key evolutionary and ecological genetic processes operating in natural plant populations, such as gene dispersal, natural selection and genetic drift. Analysis of SGS is also important for understanding the population dynamics of a species as its response to micro-environmental variation and global climate change [16,17]. Furthermore, knowledge of SGS in natural populations is also important for developing scientifically-sound sampling strategies for genetic resource conservation and breeding purposes.

Higher SGS may be expected in peripheral than in core populations of long-lived plants, such as forest trees, owing to the differences in founding population, seed and pollen dispersal, adult plant density, inbreeding levels, effective population size (*N_e_*), and disturbance and selection regimes. Restricted gene flow due to limited seed and pollen dispersal can promote mating among relatives which can result in isolation by distance within a population [18]. Therefore, a local genetic subdivision can develop SGS within populations. Selfing and other forms of inbreeding can limit gene dispersal, consequently influencing the spatial distribution of genetic variation within populations [16]. In contrast, generally, outcrossing decreases population subdivision by reducing mating among relatives and increasing *N_e _*[10]. Therefore, higher level of SGS can be expected in inbreeding species as compared to outcrossing species. Vegetative clonal reproduction can significantly affect the SGS through the development of spatial clusters of the members of a clone [19]. Long-lived plants are expected to have low levels of SGS within their populations because of decreased population divergence due to increased *N_e _*and decreased genetic drift over time [10]. SGS has been found to be inversely correlated with adult density within populations [20]. Additionally, the levels of SGS were found to be positively correlated with the level of fragmentation of plant populations e.g., [6,14,21].

Conifers are generally long-lived plants that are ecologically and economically important globally. They constitute a major component of the northern boreal and temperate forests, especially dominating the boreal forest which comprises one of the largest biomes in the world http://www.borealforest.org/index.php?category=world_boreal_forest&page=overview. Most of the conifer tree species are characterized by predominantly (~90%) outcrossing mating system. Studies in conifers reported either no or weak levels of SGS within populations [21-32]. This is consistent with the conifers' characteristics of predominant outcrossing, wind-pollination and seed dispersal by wind. Except for two studies [24,32], all of the SGS studies in conifers are reported for *Pinus *or *Picea *species. Furthermore, with the exception of two [8,28], all other studies on SGS in conifers are based on only one or two populations. All of the SGS studies in conifers have been reported for predominantly outcrossing (≥90%) species, and there is no SGS information on plant species that have mixed-mating system with high selfing levels.

Eastern (or northern) white cedar (*Thuja occidentalis *L.) provides an ideal model species to study SGS in core versus peripheral populations of declining and long-lived plant species with mixed mating system. Eastern white cedar is a wind-pollinated, monoecious, evergreen tree species belonging to the Cypress family (Cupressaceae). It reproduces both sexually and vegetatively. Vegetative reproduction occurs mainly in swampy sites by layering [33]. Eastern white cedar is an ecologically and economically important tree species, highly suitable for many wood products. Since its foliage is a favorite winter food for deer, it is very important for wildlife conservation. It can reach an age of 400 years [34].

**Figure 1:**
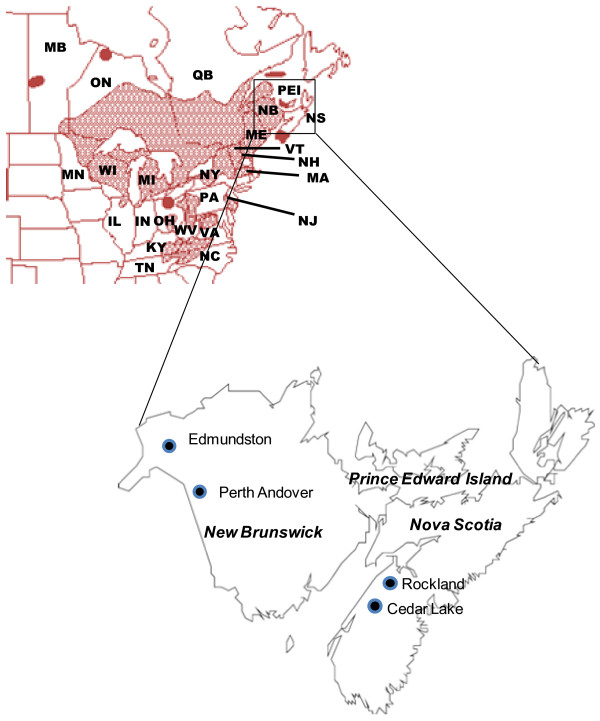
**A map showing the geographical distribution [from: http://www.efloras.org/object_page.aspx?object_id=5261&flora_id=1] (above) of *Thuja occidentalis *and approximate locations of its four studied populations (below) (see also Table 1)**.

The natural distribution range of eastern white cedar extends from Manitoba east to Nova Scotia, and south into the Great Lakes Regions of the United States, east to New England, with southern disjunct populations in the Appalachians in North Carolina and Tennessee ([34]; Figure 1). Eastern white cedar reaches its eastern periphery in Nova Scotia and the Prince Edward Island (PEI), where it occurs in small, scattered and fragmented populations [34,35]. Historical records indicate that the range and number of populations of eastern white cedar have diminished due to logging, and clearing for agriculture over the past 400 years in Nova Scotia and PEI [33,35]. Presently, there are only 32 identified populations of eastern white cedar in Nova Scotia [34], which are highly fragmented. Due to its rarity in Nova Scotia, eastern white cedar has been designated as a Red (endangered) species in this province. In Prince Edward Island, occurrence of eastern white cedar is limited to only a few populations, mainly found in the western part of the Island (West of Summerside). Eastern white cedar is common in New Brunswick, where it forms its best development [35]. Thus, New Brunswick represents the core or central range of this species. However, here also eastern white cedar has been listed as a species of concern.

Like other cedar species (*Thuja plicata, T. orientalis*, [36-38]), eastern white cedar also possesses relatively low genetic diversity [39-41]. It is characterized by a mixed-mating system with high levels (mean 54%) of selfing rates [41,42]. Although a few genetic diversity studies have been conducted in the genus *Thuja *[36-41,43], to our best knowledge, there is no information available on SGS in any *Thuja *species. The only SGS study in the Cupressaceae family was done for one population of an outcrossing species, *Cryptomeria japonica *[32], which reported significant SGS at short distance classes (up to 60 m) based on nuclear microsatellites, but non-significant SGS for chloroplast microsatellites.

The aim of the present study was to determine within-population fine-scale genetic structure of eastern white cedar growing in core and peripheral populations. Our hypotheses were: (i) since eastern white cedar is a mixed-mating long-lived tree with high levels of selfing rate, and has capability to reproduce vegetatively, this species is expected to have some levels of SGS within population; (ii) the peripheral populations of eastern white cedar are expected to have higher levels of SGS than the core populations due to their lower population density; potentially restricted gene dispersal, smaller *N_e_*, and higher anthropogenic fragmentation as compared to the core populations [9-13].

## Methods

### Populations and sampling

Four natural populations of eastern white cedar were sampled from two Maritime Provinces of Canada. Two of these were core (Edmundston and Perth Andover) populations from New Brunswick and other two were peripheral (Cedar Lake and Rockland) populations from Nova Scotia (Figure 1; Table 1). We restricted sampling of the core and peripheral populations to the same Maritime region of eastern Canada to avoid other regional and ecological effects confounding the results. The two populations sampled in New Brunswick are located in the central part of the species' range. Despite some harvesting activities, eastern white cedar populations remain intact with contiguous distribution in this province with no barrier to gene flow between populations. On the other hand, the two eastern white cedar populations sampled from Nova Scotia are disjunct, and highly fragmented and disturbed as they have experienced anthropogenic disturbances over a long period of time. It was not possible to find undisturbed pristine natural populations of eastern white cedar in both core and peripheral regions in eastern Canada. Also, fragmented core and contiguous peripheral populations could not be found in our study region. All of the sampled populations are over 100 years old. The populations sampled in Nova Scotia were from almost pure eastern white cedar stands, whereas the populations sampled from New Brunswick are mixed with other tree species, such as black spruce (*Picea mariana*) and balsam fir (*Abies balsamea*).

**Table 1:** Geographical location, sample size and stand density of four sampled populations of eastern white cedar

Province	Population	Code	Range location	Latitude (N)	Longitude (W)	No. of individuals sampled	Stand density (Trees/ha)
New Brunswick	Edmundston	ED	Core	47° 33' 16″	68° 13' 53″	100	46.72
New Brunswick	Perth Andover	PA	Core	46° 44' 53″	67° 42' 34″	100	58.82
Nova Scotia	Cedar Lake	CL	Peripheral	44° 50' 22″	65° 01' 48″	100	18.20
Nova Scotia	Rockland	RL	Peripheral	45° 00' 35″	64° 42' 10″	94	24.00

**Figure 2:**
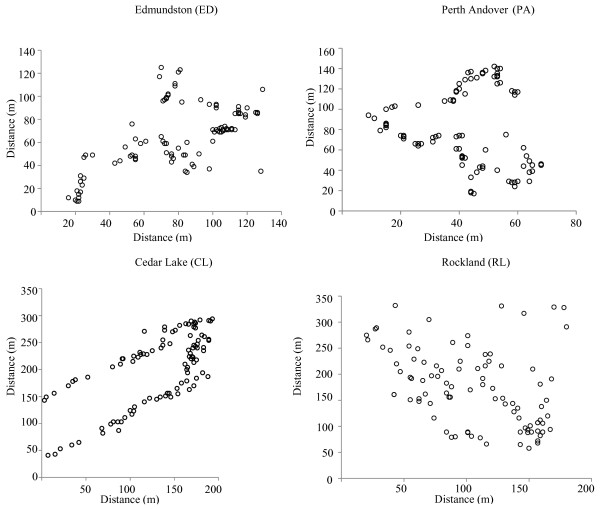
**Spatial distribution of individual trees in each of the four studied eastern white cedar populations**.

A plot consisting of ~ 100 contiguous eastern white cedar trees was established in each of the four sampled populations irrespective of the plot area (Figure 2). All of the eastern white cedar individuals were sampled within these plots. We chose this sampling strategy to keep the sample size equal among all four sampled populations, in order to minimize the effects of sample size confounding the comparison of SGS results between the populations. Spatial locations of each of the sampled trees were recorded as their geo-coordinates using a Geographical Positioning System (Garmin GPS MAP 60CSx). The spatial location maps of the sampled trees in each of the four populations are provided in Figure 2.

Small amounts of foliage samples were collected from each of the 394 sampled trees in plastic Ziplock bags containing silica gel. Upon arrival to the lab, the foliage samples were stored at -20°C until processed for DNA extraction.

### DNA extraction and genotyping

Total genomic DNA was extracted from the needle samples of individual trees using the DNeasy Plant Mini Kit (Qiagen, USA). Microsatellite DNA markers of the nuclear genome were used for genotyping the sampled trees. A total of 40 microsatellite markers developed for the Cupressaceae species were tested in eastern white cedar. Of these, 37 were developed for *Thuja plicata *[44,45], [L.M. O'Connell & C.E. Ritland, Unpublished data; C.J. Newton, Unpublished data] and three for *Chameacyparis obtusa *[46]. Only six (TP1, TP2, TP4, TP6, TP11 and 91 G) of the 40 microsatellites yielded simple and scorable PCR (Polymerase Chain Reaction) products. These six microsatellites were optimized and used for genotyping individual eastern white cedar trees. PCR amplifications were carried out using 10 μl reaction mixtures containing 20 to 30 ng of genomic DNA, 1× PCR buffer, 1.5 mM MgCl_2_, 0.20 mM of each dNTP, 0.50 pmol of each primer and 0.25 U *Taq *DNA polymerase (Invitrogen, USA). The epGradients Master Cycles (Eppendorf, Germany) were used for PCR amplification of the sample DNA, with the following thermocycling profile: initial denaturation step at 95°C for 5 min, followed by 33 cycles each of 95°C for 45 sec, annealing temperature at 55 to 65°C for 45 sec, 72°C for 45 sec and final extension step of 72°C for 5 min. PCR products were separated on LI-COR4300 genetic analyzers (LI-COR Inc. Lincoln, Nebraska, USA). The genotypes of individual trees were first determined by scoring the LI-COR gels with the SAGA GT program (LI-COR Inc. Lincoln, Nebraska, USA) and later verified by manual scoring.

### Statistical data analysis

#### Genetic diversity and N_e_

The standard population genetic diversity parameters (number of alleles, observed and expected heterozygosity) and fixation index (*F*) were calculated using the program GENALEX [47]. Allelic richness (A_R_) was estimated using the program FSTAT [48]. Genotypic diversity was determined by calculating observed and expected genotype additivity (GA_O _and GA_E_) as the sum of genotypes observed and expected under Hardy-Weinberg equilibrium over the loci [49]. Deviations of genotypic frequencies from the Hardy-Weinberg expectation were tested using the Markov chain method (parameter values: dememorisation number = 10,000, number of batches = 300, number of iterations per batch = 5,000) in the GENEPOP program [50].

The effective population size (*N_e_*) for each of the four populations was estimated using the maximum likelihood method of coalescent-theory and MIGRATE program [51]. At first, the θ values were estimated for each population using MIGRATE program and assuming an average microsatellite mutation rate of 10^-3 ^per generation. The *N_e _*was calculated as θ/(4×10.^-3^).

#### Spatial distribution

Pattern of spatial distribution of individual trees in the four eastern white cedar populations were estimated using univariate second-order Ripley's [52]*K*-function for 10, 15, 20 and 30 m distance classes (t). The weighted edge correction [53] method was applied to correct the edge effect. Monte Carlo procedure was used to test the significance of deviation from a random distribution at 95% upper and lower confidence envelops, generating 10,000 replications. These calculations were performed using the SPPACK program [54].

#### Spatial Genetic Structure (SGS)

We used Smouse and Peakall's [12] spatial autocorrelation method to estimate the SGS in the four eastern white cedar populations employing the GENALEX 6.2 [47] program. This method determines spatial distribution of genetic variation based on a frequency-weighted average over all alleles and loci and correlation coefficients of genotypes in relation to their spatial distance. It allows for the covariance between alleles (see [12] for detailed description). In order to facilitate the comparison of SGS among the four populations, we used 15 m distance class as representative because three, out of the four populations showed maximum values of Ripley's *K *at this distance class. Similar approach was used by Oddou-Muratorio et al. [55] to select the distance class for SGS analysis in a tree species, *Sorbus torminalis*.

In addition to the estimation of multilocus SGS parameters for each of the four populations separately, SGS analyses were also performed for combined subset each of core (ED, and PA), and peripheral (CL and RL) populations, as well by combining all four populations to infer degrees of heterogeneity of SGS within and between subsets based on the method of Smouse et al. [56] using GENALEX 6.2 program. These analyses permit us to formally test whether differences in SGS between the subsets of populations are statistically significant. Since the analysis of SGS using combined multiple populations increases sample sizes, it increases statistical power of the analysis to detect the presence of SGS within populations. Heterogeneity tests for single and multi-populations correlograms were performed as described by Smouse et al. [56]. This method first performs the heterogeneity test of autocorrelation coefficient among individuals lag-by-lag using the pooled within-population correlograms. Then the single-lag tests are used to perform an analogous heterogeneity test of multilag correlograms (see Smouse et al. [56] for detailed description). Single class (*t^2^*) and multi-class (ω) test criteria were used to test the heterogeneity of spatial autocorrelation (*r*) values between single and subsets of populations. Heterogeneity in SGS for single distance classes between any two populations or a subset of populations was determined by performing the squared paired- sample *t *test (*t*^2^) [56]. SGS heterogeneity over all distance classes (a) in a population and (b) among populations was determined by using the ω test criteria [56] under the null hypotheses that (a) there is no significant spatial autocorrelation at any distance class, and that (b) the spatial correlograms from all populations are homogeneous. Statistical significance of all the analyses was performed by permutation test with 9,999 replications at the 95% upper and lower confidence intervals.

The correlations of the SGS parameters (significant spatial autocorrelation coefficient, *r*) with *N_e _*(actual or log transformed), genetic diversity parameters and density of eastern white cedar trees within the sampled populations were determined by calculating the Pearson correlation coefficients (R), using the MINITAB 15 package.

### *Sp *statistics and dispersal estimates

In order to quantify SGS and to compute neighborhood sizes and gene dispersal distances in the four eastern white cedar populations, *Sp *statistic was estimated as follows: *Sp *= -^^^b_F_/(1-^^^*F_1_*), where -^^^b_F _is the regression slope of the autocorrelogram and *^^^F_1 _*is the mean kinship coefficient (*Fij*) [57] between individuals belonging to the first (0-15 m) distance class [21]. Using the *Sp *statistics, Wright's neighborhood size *Nb *(*Nb *= 1/*Sp*) was also estimated. *Nb *is defined as *Nb *= 4π*D_e_*σ_g_^2^, where σ_g_^2 ^is the axial variance of gene dispersal and *D_e _*the effective population density. Estimates of gene dispersal distance (σg) were determined assuming ratios of effective to census density, *De/D*, of 0.1 and 0.5 [7,21]. Estimates of -^^^b_F _and *^^^F_1 _*were carried out using SPAGEDI program [58]. Statistical significance (*P *≤ 0.05) of the analyses was performed by permutation tests with 10,000 replications.

## Results

### Genetic diversity and effective population size

In total, 95 alleles were observed at six microsatellite loci in four eastern white cedar populations. The core populations showed higher allelic as well as genotypic genetic diversity than the peripheral populations (Table 2). Also, the core populations possessed higher number of private alleles than the peripheral ones (Table 2). The two core populations from New Brunswick (ED and PA) showed very similar levels of genetic diversity. All four populations showed significant deviation from the Hardy-Weinberg equilibrium. The *N_e _*was 432 and 910, with an average of 671 in the core populations, and 110 and 310, with an average of 210 in the peripheral populations (Table 2). As the objective of this study was to determine SGS, we do not focus on discussing genetic diversity results here. Results on genetic diversity and population structure of 13 eastern white cedar populations from the same region are presented and discussed elsewhere [59].

**Table 2:** Genetic diversity measures, fixation index and their (SE), and effective population size for four sampled populations of eastern white cedar

Pop type	Province	Population	**A**_**T**_	A	**A**_**E**_	**A**_**R**_	**A**_**P**_	**H**_**O**_	**H**_**E**_	**GA**_**O**_	**GA**_**E**_	*F*	*N_e_*
Core	New Brunswick	ED	63.00	10.50 (1.86)	4.19 (1.15)	10.08 (2.09)	11	0.640 (0.057)	0.662 (0.079)	127 (3.84)	404 (7.43)	0.007 (0.062)	432
		PA	67.00	11.17 (2.63)	4.68 (1.51)	10.68 (2.51)	9	0.640 (0.087)	0.640 (0.103)	145 (4.53)	500 (9.03)	-0.025 (0.048)	910
Peripheral	Nova Scotia	CL	53.00	8.83 (2.02)	2.79 (0.44)	8.42 (2.15)	6	0.571 (0.070)	0.597 (0.060)	100 (3.12)	312 (6.69)	0.050 (0.034)	310
		RL	47.00	7.83 (2.17)	2.60 (0.48)	7.66 (2.27)	5	0.505 (0.111)	0.519 (0.110)	77 (2.94)	267 (7.07)	0.044 (0.048)	110
	Mean	Core	65.00	10.83 (2.24)	4.43 (1.33)	10.38 (2.30)	10	0.640 (0.072)	0.651 (0.091)	136.00 (4.19)	452 (8.23)	-0.009 (0.055)	671
		Peripheral	50.00	8.33 (2.10)	2.70 (0.46)	8.04 (2.21)	5.50	0.540 (0.091)	0.560 (0.085)	88.50 (3.03)	289.50 (6.88)	0.047 (0.041)	210
	Overall mean		57.50	9.58 (2.17)	3.57 (0.89)	9.21 (2.26)	7.75	0.590 (0.082)	0.600 (0.088)	112.25 (3.61)	370.75 (7.55)	0.019 (0.048)	440

A_T_, total number of alleles; A, mean number of alleles per locus; A_**E**_, effective number of alleles; A_R_, Allelic richness; A_P_, number of private alleles; GA_O_, observed genotype additivity (richness); GA_E_, expected genotype additivity [49]; H_**O**_, observed heterozygosity; H_**E**_, expected heterozygosity; *F*, fixation index; *N_e_*, effective population size. Values in parentheses are standard errors.

ED, Emundston; PA, Perth Andover; CL, Cedar Lake; RL, Rockland

### Spatial distribution

**Figure 3:**
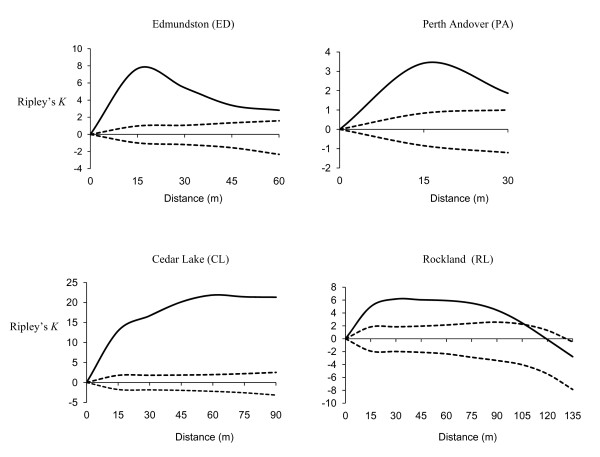
**Patterns of spatial distribution of individual trees in four eastern white cedar populations using Ripley's *K *function (solid line), dashed lines represent 95% confidence envelope**.

Results of spatial distribution pattern analysis using Ripley's *K*-function and the number of pairs of observation for 15 m distance class are presented in Figure 3, and Table 3, respectively. All four populations showed significant aggregation of individuals at the distance classes 10, 15, 20 and 30 m (results are shown only for 15 m distance class).

**Table 3:** Number of pairs of observation per distance class for the four eastern white cedar populations

Distance class (m)	Population			
	
	ED	PA	CL	RL
0-15	581	651	231	122
16-30	768	1014	345	262
31-45	919	700	430	327
46-60	1025	737	473	385
61-75	701	816	469	427
76-90	307	550	567	432
91-105	303	344	435	399
106-120	249	126	324	371
121-135	89	12	308	381
136-150	8		271	301
151-165			209	217
166-180			203	181
181-195			158	154
196-210			124	100
211-225			96	116
226-240			63	85
241-255			49	63
256-270			41	38
270-285			31	7
286-300			17	3
301-315			7	

ED, Emundston; PA, Perth Andover; CL, Cedar Lake; RL, Rockland

### Spatial genetic structure (SGS)

**Figure 4:**
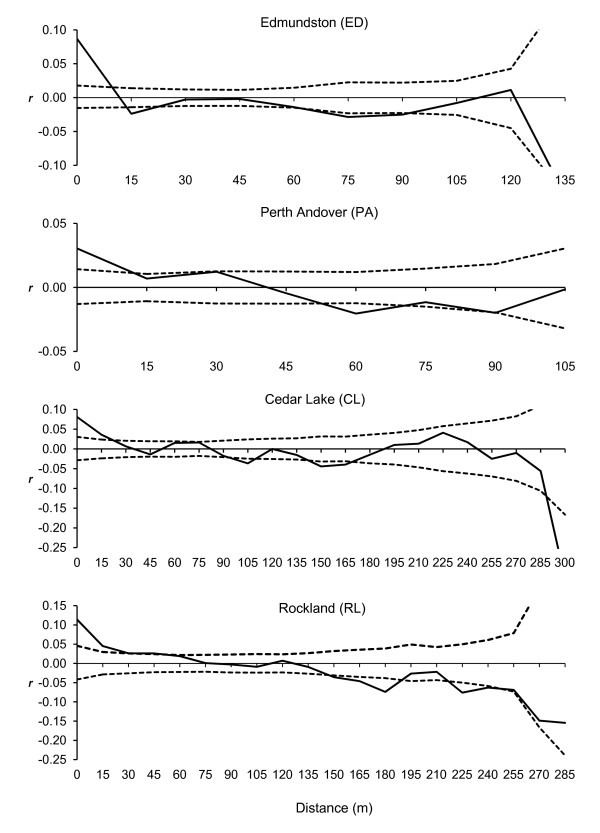
**Correlograms of spatial autocorrelation coefficients (*r*) (solid lines) plotted against geographical distance classes, estimated for four eastern white cedar populations**. Dashed lines represent upper and lower 95% confidence limits.

Results of the multilocus spatial genetic structure (SGS) analysis based on Smouse & Peakall's [12] spatial autocorrelation coefficient (*r*) are presented in Figure 4. All four studied populations showed positive significant SGS in short distance classes ranging from 15 m in ED and PA to 75 m in RL (Figure 4). Analysis of the combined core (ED and PA) populations showed significant SGS up to 15 m, whereas analysis of the combined peripheral (CL and RL) populations showed significant SGS up to 90 m (Table 4). An overall significance test (ω) of spatial autocorrelations and correlograms revealed that all single or combined-populations were statistically significant (Table 4).

**Table 4:** Spatial autocorrelation coefficients (*r*), single-class squared pair-sample *t-*test (*t*^2^) and multi-class and multi-population (ω) heterogeneity tests of spatial autocorrelation coefficients (*r*) for individual populations, population pairs and subsets of populations

Populations	*r *and *t^2 ^*values and their statistical significance in different distance classes								Overall multi-distance class or multi population correlogram significance (ω-test criterion)
	**0-15**	**16-30**	**31-45**	**46-60**	**61-75**	**76-90**	**91-105**	**106-120**	
	Spatial autocorrelation coefficients (*r*)								
ED	0.087*	-0.024	-0.003	-0.002	-0.014	-0.029	-0.025	-0.008	75.35*
PA	0.030*	0.007	0.012	-0.005	-0.020	-0.012	-0.020	-0.002	70.49*
CL	0.081*	0.036*	0.006	-0.014	0.015	0.016	-0.017	-0.037	134.39*
RL	0.114*	0.045*	0.026*	0.026*	0.019*	0.001	-0.003	-0.009	147.32*
Core	0.058*	-0.007	0.003	-0.003	-0.017	-0.018	-0.022	-0.006	81.11*
Peripheral	0.093*	0.040*	0.016*	0.005*	0.017*	0.009*	-0.010	-0.022	154.80*
Four pops combined	0.066*	0.005	0.007*	0.000	-0.004	-0.003	-0.015	-0.016	65.65*
	Single-class squared pair-sample *t-*test (*t*^2^)								
ED vs. PA	17.53*	8.44*	1.89	0.06	0.37	1.13	0.09	3.89	47.99*
ED vs. CL	0.10	17.13*	0.52	0.96	5.50*	8.03*	0.21	0.16	51.66*
ED vs. RL	1.34	18.83*	4.25*	4.70*	6.67*	3.07	1.71	0.02	57.02*
PA vs. CL	7.82*	4.54*	0.18	0.52	8.69*	4.31*	0.02	3.57	49.70*
PA vs. RL	12.76*	6.15*	0.93	5.05*	10.11*	0.72	1.03	4.00	62.34*
CL vs. RL	1.55	0.24	1.48	7.07*	0.07	1.20	0.85	0.16	28.08
Core vs. peripheral	4.72*	18.64	1.58	0.73	15.08*	6.83*	1.04	1.23	65.26*

ED, Edmundston; PA, Perth Andover; CL, Cedar Lake; RL, Rockland. * indicates significant values (*P *≤ 0.05)

The maximum values of spatial autocorrelation coefficient (*r*) were observed for the first (0-15 m) distance class for individual or combined populations (Table 4). The *r *values for the first distance class showed great variation among the four populations ranging from 0.030 (PA population) to 0.114 (RL population) (Figure 4). Thus, *r i*n RL was about four times of that in PA. The highest *r *for the combined peripheral populations (*r *= 0.093) was considerably higher than in the intact populations (*r *= 0.058).

Except for the population pair CL and RL, all pairs revealed significant heterogeneity in SGS between the populations based on the multi-class test criterion (ω) (Table 4). Significant heterogeneity in SGS was also observed between the core and peripheral populations over the 0-15 m and 61-90 m distances (up to 90 m) (Table 4).

### *Sp *statistics and dispersal estimates

Results of *Sp *statistics and dispersal estimates in the four eastern white cedar populations are presented in Table 5. *Sp *values were 0.009 (PA) and 0.018 (ED) in the core populations, and 0.016 (CL) and 0.031 (RL) in the peripheral populations. Average *Sp *value was higher in the peripheral (0.023) than in the core (0.014) populations Average value for Wright's neighborhood size (*Nb*) was higher in the core populations (81.57) as compared to the peripheral populations (48.04). Gene flow distances (σ_g_) were 44 and 120 m in the core populations and 46 and 167 m in the peripheral populations (Table 5).

**Table 5:** Spatial genetic structure, Sp statistics and estimates of gene flow distances in four eastern white cedar populations

Region	Population	*^^^F1*	*^^^F2*	*^^^F3*	*^^^F4*	*^^^F5*	b-log (^^^bF)	*Sp*	*Nb*	σ_g _(m) (*De/D *= 0.1)	σ_g _(m) (*De/D *= 0.5)
Core	ED	0.047*	-0.013*	-0.001	-0.001	-0.008	-0.017*	0.018	56.73	98.29	43.96
	PA	0.015*	0.004	0.006	0.008	-0.013	-0.009*	0.009	106.41	119.98	53.66
Peripheral	CL	0.045*	0.023*	0.003	-0.008	-0.005	-0.015*	0.016	64.00	167.32	74.83
	RL	0.070*	0.027*	0.026*	0.015*	0.014*	-0.029*	0.031	32.08	103.14	46.13
Average core		0.031	0.004	0.003	0.004	-0.011	-0.013	0.014	81.57	109.14	48.81
Average peripheral		0.057	0.025	0.015	0.004	0.005	-0.022	0.023	48.04	135.23	60.48

*^^^F1, ^^^F2, ^^^F3, ^^^F4 *and *^^^F5*: mean kinship coefficient (*Fij*) [57] between individuals belonging to the first (0-15 m), second (15-30 m), third (30- 45 m), fourth (45-60 m) and fifth (60-75 m) distance classes, respectively; b-log (^^^bF): Slope of the regression of kinship with ln(dist); *Sp*: statistic defined by the ratio _^^^bF/(1 - *^^^F1*), where ^^^bF is the regression slope of the autocorrelogram; *^^^F1 *is the mean kinship coefficient (*Fij*) between individuals belonging to the first (0-15 m) distance class [20]; *Nb *Wright's neighbourhood size; σg, estimate of gene flow distance from *Sp*, assuming different ratios of effective (*De*) to census density (*D*); *, significant (*P *< 0.05)

ED, Edmundston; PA, Perth Andover; CL, Cedar Lake; RL, Rockland

### Correlations of SGS parameters with genetic diversity, stand density, and population *Ne*

There was negative correlation of the significant SGS (spatial autocorrelation coefficient *r*) with the population genetic diversity measures. The correlations (Pearson correlation coefficient R) of *r *with genetic diversity measures were as follows: mean number of alleles per locus (A) = -0.85 *P *= 0.034), observed heterozygosity (H_O_) = - 0.89 (*P *= 0.022), expected heterozygosity (H_E_) = -0.82 (*P *= 0.044), observed genotypic richness (GA_O_) = -0.88 (*P *= 0.024) and expected genotypic richness (GA_E_) = 08 *P *= 0.040). The correlation of eastern white cedar stand density with *r *a -0.69 *P *= 0.096). The significant SGS *r *values showed negative high correlation (Pearson R = -0.65)wt *Ne*, although the correlation was not statistically significant (*P *= 0.139).

## Discussion

### Spatial genetic structure in eastern white cedar

Our study demonstrates that eastern white cedar has significant within-population fine-scale genetic structure at short distances. This was clearly evident from significant positive SGS at ~15 m to ~75 m in four studied eastern white cedar populations; suggesting that individuals with similar alleles or genotypes were more often growing in proximity than expected by chance alone. The extent of the observed significant SGS depended upon the location of the populations (core versus peripheral). These results are discussed later in this section.

The extent of SGS for combined four eastern white cedar populations (up to 45 m) is more than two times of that observed in one 20-years old population of another mixed-mating (mean selfing rate of 27% [60]) species *Larix larcinia *(up to 20 m) [33]. The existence of SGS in *Larix larcinia *was explained primarily due to the effect of founding population. In contrast, predominantly-outcrossing species showed either weak or no within-population SGS e.g. [17,22,28]. Thus, mixed-mating conifer species tolerating high levels of selfing apparently have higher SGS within their populations than the predominantly outcrossing conifers.

Besides higher levels of selfing or inbreeding, limited gene flow and founding gene pool may be responsible for the observed short-distance SGS in eastern white cedar. Restricted gene flow due to limited pollen and seed dispersal contributes to local grouping of individuals with similar alleles or genotypes [10]. In eastern white cedar, both pollen and seeds are disseminated by wind. We are not aware of any information on the average distance of pollen dispersal in this species. However, in conifers it is well known that pollen is dispersed in enormous amounts over considerable distances [61,62]. Therefore, the restriction in gene flow due to limited pollen flow is less likely to happen in eastern white cedar, especially in the contiguous populations. However, in the peripheral populations, reduced gene flow may have contributed to the observed levels of fine-scale genetic structure as a result of fragmentation. Fowells [63] reported a seeding range of up to 60 m in eastern white cedar under normal conditions. Interestingly, the mean level of SGS observed in eastern white cedar (up to 45 m) falls within this range of the seeding distance. Therefore, restricted gene flow due to limited seed dispersal may be one of the main reasons for the observed short-distance SGS in the species.

Vegetative propagation through natural layering has been reported in eastern white cedar [33,64]. Since the individuals established from vegetative propagation have genetic constitution identical to the source tree and grow in proximity forming clumps, this may generate a significant SGS at short distance intervals. However, we did not find any concrete evidence for vegetative propagation contributing to the observed levels of SGS in any of the four study populations. We detected only four to eight individuals (4 to 8%) per population growing in proximity of each other with the same multilocus genotypes (data not shown). This level of multilocus genotype sharing may be due to vegetative reproduction, selfing, a combination of these factors or just by chance. However, our data does not support vegetative propagation contributing to the observed levels of SGS in eastern white cedar. Although, it is generally expected that vegetative reproduction is one of the major factors that may contribute to the occurrence of significant SGS in vegetatively reproducing species, Chung & Epperson [65] found little effect of vegetative reproduction on SGS in *Eurya emarginata*. They explained that the restriction in gene flow due to limited dispersal of seed and pollen could be the main reason for the observed SGS in the species.

### Spatial distribution pattern of trees

The analysis of spatial distribution pattern using Ripley's *K *function indicated significant aggregation of trees in all four populations of eastern white cedar. Within population spatial patterns of individual plants can be influenced by several factors such as variation in microsites, level of disturbance, seed dispersal patterns [56,66]. Parker et al. [66] and Trabaud et al. [67] reported a significant aggregation of tress in *Pinus clausa *and *Pinus halepensis *populations, respectively, and they explained that the observed aggregation could be due to either limited seed dispersal or recruitment of aggregated seedlings after stand disturbance due to fire. These factors may have contributed to the observed spatial pattern of individual trees in eastern white cedar populations. The effect of limited seed dispersal on the significant aggregation of individual trees may be evident from the significant SGS (15-75 m) observed in the four populations. Although we are not aware of any records of fire in the studied populations, past occurrence of fire on the studied populations cannot be ruled out because fire is one of the main agents of natural disturbance in boreal forest [68].

### SGS in core versus peripheral populations

The mean significant SGS in the peripheral populations (up to 90 m) was up to six times of that was observed in the core (15 m) populations. The difference in SGS between the core and peripheral populations was statistically significant (ω = 65.26, *P *= 0.001). Both Nova Scotia populations (CL and RL) showed the highest within-population SGS, and the lowest genetic diversity, stand density and *N_e_*. These populations are highly fragmented and affected by human disturbance for over 400 years ever since the arrival of European settlers in the Province. The several-folds higher SGS levels observed in the two marginal and highly fragmented populations from Nova Scotia is likely due to reduced stand density, gene dispersal, genetic diversity and *N_e _*observed in these populations (Tables 1 and 2) as well as increased founder effect and genetic drift due to anthropogenic fragmentation and other disturbances. Thus, the magnitude of evolutionary forces operating in core versus peripheral populations is probably different. It was not possible to dissect out the effects of anthropogenic fragmentation and disturbances on SGS in the peripheral populations from that of their peripheral location. Comparisons of SGS between pristine core and pristine peripheral populations would have been ideal. However, in the eastern Maritime Canadian range of eastern white cedar where our study was focused, no peripheral population could be found that is not anthropogenically fragmented and disturbed. Thus, fragmentation and disturbance may also have contributed to higher SGS observed in the peripheral populations. The negative correlations of SGS with stand density, genetic diversity parameters and *N_e _*are consistent with the population genetic theories and expectations. The negative correlation between genetic diversity and SGS could be due to the combined effect of founder effects and reduced stand density.

The spatial distribution of genotypes was found to be random in the core populations of Sitka spruce, whereas significant positive SGS up to 500 m was observed in the peripheral populations of this species [8]. In eastern white cedar, we observed mean significant positive SGS up to 90 m (combined populations) in the peripheral and up to 15 m in the core populations. Thus, the magnitude of impact of ecological and evolutionary factors on SGS in peripheral populations of Sitka spruce appears to be much higher than in eastern white cedar. Sitka spruce is a predominantly outcrossing species. The extent of differences observed in within-population SGS in core versus peripheral populations between these species may be related to the differences in their mating system. The mixed-mating species with relatively low inherent genetic diversity, such as eastern white cedar, can perhaps tolerate better negative effects (increased inbreeding, decreased genetic diversity and *N_e_*) than the predominantly outcrossing species with relatively high genetic diversity, such as Sitka spruce. However, this aspect remains to be further tested in more details globally.

### *Sp *statistics and gene dispersal distance

In agreement with the SGS results estimated using Smouse and Peakall's [12] autocorrelation coefficient method, average value for *Sp *statistic was also higher in peripheral than in core populations of eastern white cedar. Vekemans and Hardy [20] reported significantly higher levels of *Sp *value in populations with low density as compared to the populations with high density. Since the density of trees in the peripheral populations was much lower than in the core ones, the effect of density on the results of *Sp *cannot be ruled out. Except in the population PA, *Sp *values observed in the other three populations of eastern white cedar were higher than the average value observed in five wind-dispersed temperate trees (*Sp *= 0.012) and 18 outcrossing plant species [20]. However, it was lower in eastern white cedar as compared to the average value (*Sp *= 0.037) of seven mixed mating species [20]. De-Lucas et al. [7] reported higher *Sp *values in fragmented than in continuous populations of another conifer species, *Pinus pinaster*, and they explained that the lower effective population size due to few reproductive individuals in fragmented populations may have resulted in restricted gene flow via seed and increased the level of SGS in the populations. This explanation may also be applicable to eastern white cedar because the peripheral populations were highly affected by anthropogenic fragmentation. The average gene flow distance in eastern white cedar was higher in peripheral populations than in the core ones. Higher gene flow distances would be expected in fragmented and peripheral populations than in core and intact populations because of lower tree density in peripheral populations resulting in lower obstruction for pollen movement [20,69], which is the case in peripheral populations of eastern white cedar. In agreement with our results, Vekemans and Hardy [20] also reported higher gene flow distances in low density populations than in populations with high density.

### Evolutionary potential and genetic resource conservation implications

The results of genetic diversity, *N*e and SGS comparisons in core versus peripheral populations suggest that the evolutionary potential of the studied peripheral eastern white cedar populations may have been adversely affected. The results of our study have significance and implications for conservation and management of genetic resources of eastern white cedar - a declining to endangered species in Atlantic Canada. For *ex-situ *conservation via seed and vegetative propagule collection, a minimum distance of 75 meters in peripheral and 20 m in core populations is advisable to capture maximum genetic diversity and avoid sampling genetically-related trees. The core populations in New Brunswick should be conserved *in-situ*. Both of the peripheral Nova Scotia populations have suffered genetic degradation as a result of extensive human disturbance and edge effect. Here, both *ex-situ *and *in-situ *genetic resource conservation efforts supplemented with genetic restoration will be required given that eastern white cedar is a red-listed species in Nova Scotia. The results of our study are relevant for within-population sampling of individuals for population genetic studies in this species. The results may also be applicable for conservation and management of genetic resources of other long-lived, declining species with similar mixed-mating characteristics.

## Conclusions

In conclusion, our results clearly demonstrate that the studied eastern white cedar populations have significant fine-scale genetic structure at short distances. Peripheral populations have several-folds higher within-population fine-scale genetic structure than core populations. Vegetative propagation does not apparently contribute to SGS in eastern white cedar. Anthropogenic disturbances and fragmentation presumably have significant effects on fine-scale genetic structure in eastern white cedar. The magnitude of evolutionary forces such as founder effect, genetic drift, gene flow and selection, operating in core versus peripheral populations is likely to be different. This may be the first report comparing SGS in intact core versus fragmented peripheral populations of a long-lived, mixed-mating declining plant. The results of our study contribute to the knowledge of poorly-understood spatial genetic structure of core versus peripheral populations in plants.

## Abbreviations

A_T_: Total number of alleles; A: Mean number of alleles per locus; A_**E**_: Effective number of alleles; A_R_: Allelic richness; A_P_: Number of private alleles; b-log (^bF): Slope of the regression of kinship with ln(dist); CL: Cedar Lake; D: Census density; *De: *Effective density; ED: Emundston; *F*: Fixation index; *Fij: *Mean kinship coefficient; GPS: Geographical positioning system; GA_O_: Observed genotype additivity (richness); GA_E_: Expected genotype additivity; H_O_: Observed heterozyogosity; H_E_: Expected heterozygosity; *Nb*: Wright's neighbourhood size; *Ne*: Effective population size; PA: Perth Andover; *r*: Spatial autocorrelation coefficients; RL: Rockland; *Sp*: Sp statistics; σg: Estimate of gene flow distance from *Sp*; SGS: Spatial genetic structure

## Authors' contributions

Both authors have contributed significantly to the work reported in the manuscript. MP was a Postdoctoral Fellow, who generated 50% of the eastern white cedar genotype data, performed data analysis, and drafted the manuscript. OPR is the principal investigator of the project and contributed to the conception of the study and its experimental design, provided overall guidance and direction and funding, and revised the manuscript. Both authors have read and approved the final manuscript.
